# Fundus albipunctatus

**DOI:** 10.11604/pamj.2016.23.61.8562

**Published:** 2016-02-29

**Authors:** Rajaa Elhannati, Hicham Tahri

**Affiliations:** 1 Service d'Ophtalmologie, CHU Hassan II, Fès, Maroc

**Keywords:** Fundus albipunctatus, recessive autosomal disease, night blindness

## Image in medicine

Fundus albipunctatus is a recessive auto somal disease. It is a rare form of apparently stationary night blindness characterized by the presence of myriad symmetrical round white dots in the fundus with a greater concentration in the midperiphery. The fundus shows a multitude of subtle, tiny yellow-white spots at the posterior pole, sparing the fovea and extending to the periphery (A et B). The retinal blood vessels, optic disc, peripheral fields and visuel acuity remain normal. Fluoresce in angiography shows mottled hyperfluorescence exceptat the fovea (C et D). Differential diagnosis are the retinitis punctat a albescens and the Oguchi disease. No effective treatment is available to restore full receptor cell function however, high oral doses of beta-carotenemay lead to an improvement in night blindness.

**Figure 1:**
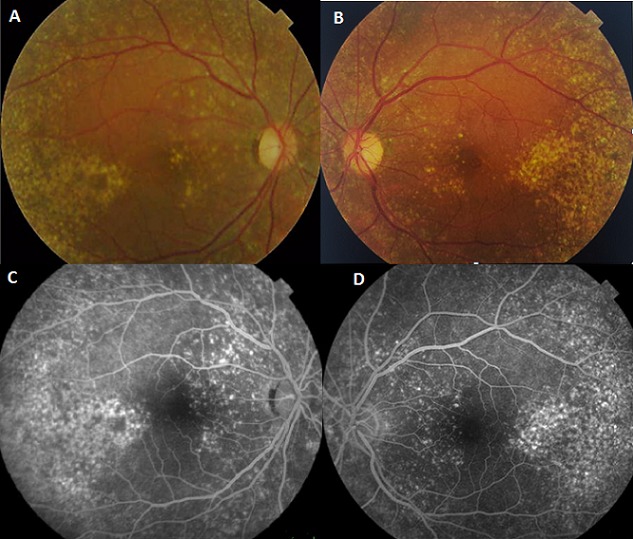
A) fundus of the right eye: yellow white spots at the posterior pole separing the fovea and extending to the periphery; B) fundus of the left eye: yellow white spots at the posterior pole separing the fovea and extending to the periphery; C) fluoresce in angiography (right eye) showed diffuse alterations of the pigment epithelium and hyperfluorescent points white spots pigment epithelium; D) fluoresce in angiography (left eye) showed diffuse alterations of the pigment epithelium and hyperfluorescent points white spots pigment epithelium

